# Nano- and microsized zeolites as a perspective material for potentiometric biosensors creation

**DOI:** 10.1186/s11671-015-0768-8

**Published:** 2015-02-11

**Authors:** Oleksandr O Soldatkin, Margaryta K Shelyakina, Valentyna N Arkhypova, Esin Soy, Salih Kaan Kirdeciler, Berna Ozansoy Kasap, Florence Lagarde, Nicole Jaffrezic-Renault, Burcu Akata Kurç, Alexei P Soldatkin, Sergei V Dzyadevych

**Affiliations:** Laboratory of Biomolecular Electronics, Institute of Molecular Biology and Genetics, National Academy of Sciences of Ukraine, Zabolotnogo Street 150, 03143 Kyiv, Ukraine; Taras Shevchenko National University of Kyiv, Volodymyrska Street 64, 01601 Kyiv, Ukraine; Central Laboratory, Middle East Technical University, 06531 Ankara, Turkey; Institute of Analytical Sciences, 5 rue de la Doua, 69100 Villeurbanne, France; Micro and Nanotechnology Department, Middle East Technical University, 06531 Ankara, Turkey

**Keywords:** Zeolite, Clinoptilolite, Zeolite BEA, Zeolite A, Enzyme, Biosensor, pH-ISFET

## Abstract

A number of potentiometric biosensors based on coimmobilization of enzymes with different types of zeolite on pH-ion-sensitive field-effect transistor (ISFET) have been developed. Their working characteristics have been determined and compared.

It was shown that clinoptilolite and zeolite Beta polymorph A (BEA) are more promising for creating biosensors than zeolite A. Changing the concentration of zeolite BEA in membranes, it is possible to extend the biosensor linear measurement range. The two-layer method of deposition of the enzyme with clinoptilolite was found to provide a significant increase in the biosensor sensitivity to substrates, whereas thermal modification of the zeolite BEA crystals can improve analytical characteristics of potentiometric biosensors for detection of toxic substances.

These results show that it is possible to regulate the ISFET characteristics for different enzyme-based biosensors by tailoring the electrode surfaces via different zeolites. This makes zeolites strong candidates for integration into biosensors as ISFET modifiers.

## Background

Immobilization of biological material is a problem of prime importance for the biosensor creation. Successful immobilization directly influences key characteristics of biosensors (activity of biological material, linear working range, sensitivity to substrate, reproducibility and operational stability of signals, duration of storage, etc.). Therefore, selection of main parameters of the immobilization process is an essential challenge in the development of biosensors. In particular, an improvement of the methods of biomembrane immobilization onto the sensing element is an important task. One of the trends for solving this problem is an application of adjuvant substances (that are carriers of biomaterial, stabilizing agents, mediators, etc.) during immobilization process.

In the literature, there are various reports about physical adsorption of enzyme on the transducer solid substrates with some supporting materials such as sol-gels [1,2], polymeric membranes [3,4] and microcapsules [5,6], nanotubes [7,8], and zeolites [9-12]. The goal of this work was to study the controlled influence of zeolite crystals on the function of potentiometric biosensors.

The use of zeolites in combination with enzymes is of considerable interest due to such characteristics of zeolites as an ability to change the surface groups, hydrophilic/hydrophobic properties, geometry, surface charge, crystal and pore sizes, and capability of acidity regulation. Additionally, zeolites are stable at high temperatures, insoluble in organic solvents, and resistant to harsh conditions of the experiment. Thus, zeolites can be used to control the microenvironment of enzymes [13]. Accordingly, the potential advantages of zeolite integration can be studied at the development of novel biosensors with improved analytical characteristics using coimmobilization of enzymes and zeolites on the surface of relevant transducers.

Zeolites can be embedded into the bioselective elements to improve analytical characteristics of biosensors, i.e., their sensitivity to the substrate, linear and dynamic ranges, and inter- and intra-reproducibility of signals. As shown in [14,15], zeolites of various kinds can be effectively used for the glucose oxidase immobilization when developing glucose amperometric biosensors to optimize their sensitivity, selectivity, and stability. As reported in [10,16,17], zeolites are used as alternative carriers for the enzyme immobilization at the creation of conductometric biosensors. Diverse variants of coimmobilization of the enzyme onto the surface of conductometric transducers were analyzed with respect to the improvement of analytical characteristics of biosensors.

In this paper, we studied and analyzed the use of a number of zeolites to improve performance characteristics of the biosensors based on pH-ion-sensitive field-effect transistor (ISFET).

## Methods

### Materials

In the work, the following enzymes were used for biosensor creation: urease (EC 3.5.1.5) from *Canavalia ensiformis*, activity 66.3 U/mg (Fluka, Switzerland) and activity 22 U/mg and 35 U/mg (Sigma, Switzerland) and butyrylcholinesterase (EC 3.1.1.8) from equine serum, activity 13 U/mg and activity 20 U/mg (Sigma, Germany). Glycerol, bovine serum albumin (BSA; fraction V), 50% aqueous solution of glutaraldehyde (GA), sodium and potassium chlorides, urea, and butyrylcholine chloride were from Sigma-Aldrich Chemie (Steinheim, Germany). Potassium-phosphate buffer (KH_2_PO_4_-K_2_HPO_4_) and NaOH were produced by Helicon (Moscow, Russia) and Sigma-Aldrich Chemie (Steinheim, Germany). Glycoalkaloids α-chaconine (purity 95%) and α-solanine (purity 95%) from potato sprouts were purchased from Sigma-Aldrich Chemie GmbH (Steinheim, Germany) and used as inhibitors for BuChE. Mercury nitrate (Hg(NO_3_)_2_) from Himlaborreaktiv (Kiev, Ukraine) was used as an activity inhibitor for urease. Other inorganic substances were of analytical grade (>98%).

### Synthesis and ion exchange procedure of zeolite crystals

Zeolite Na^+^-BEA samples were hydrothermally synthesized from gel solutions having the compositions of 1,92Na_2_O:Al_2_O_3_:ySiO_2_:4,6(TEA)_2_O:444H_2_O. The composition was determined according to the changing SiO_2_/Al_2_O_3_ ratio (*y* = 30, 40, 50, and 60) of the Na^+^-BEA samples. Sodium aluminate precursor solution for zeolite Beta polymorph A (BEA) was prepared by dissolving NaOH (>97 wt%, J.T.Baker) and sodium aluminate (50.8 wt% Al_2_O_3_ and 43.4 wt% Na_2_O, Riedel-de Haën) in deionized water (resistivity > 18 MΩ cm). Then, tetraethyl ammonium hydroxide solution (TEAOH; 35 wt%, in water, Sigma-Aldrich, Steinheim, Germany), which is the structure directing agent for zeolite BEA synthesis, was added, and the prepared precursor solution was stirred thoroughly. LUDOX® HS-40 colloidal silica solution (40 wt% SiO_2_ suspension in water, Sigma Aldrich, Steinheim, Germany) was added into aluminate precursor and mixed thoroughly before putting into the Teflon-lined stainless steel autoclaves. The autoclaves were kept statically at 120°C in a conventional oven for 7 days. The resulting solid particles were vacuum-filtered, washed with deionized water, and dried at room temperature.

The NH_4_^+^ forms of BEA crystals were obtained by ion-exchange with 1 M aqueous ammonium nitrate at 80°C for 24 h. The acid (H^+^) forms were made by calcination of NH_4_^+^-BEA 30 crystals at 500°C for 6 h.

Zeolite A was synthesized using sodium metasilicate pentahydrate (Na_2_O · SiO_2_ · 5H_2_O, 29 wt% Na_2_O, 28 wt% SiO_2_, and 43 wt% H_2_O, Fluka AG) and sodium aluminate as silicate and aluminate sources, respectively. These solutions were put into polypropylene bottles and kept statically at the designated temperatures (Table 1). The crystals were obtained by following vacuum-filtering, washing, and drying steps as in the synthesis of zeolite Beta.

**Table 1:** **Molar compositions, synthesis conditions, Si/Al ratio, and particle diameters of zeolite samples**

**Zeolite type**	**Composition**	**Synthesis conditions**	**Si/Al ratio (crystal)a**	**Particle size (μm)b**
BEA-30	1,92Na_2_O:Al_2_O_3_:30SiO_2_:4,6(TEA)2O:444H_2_O	120°C, 7 days	9.7 ± 1.0	Approximately 0.4
BEA-40	1,92Na_2_O:Al_2_O_3_:40SiO_2_:4,6(TEA)2O:444H_2_O	120°C, 7 days	13.5 ± 1.4	Approximately 0.4
BEA-50	1,92Na_2_O:Al_2_O_3_:50SiO_2_:4,6(TEA)2O:444H_2_O	120°C, 7 days	17.7 ± 1.8	Approximately 0.4
LTA-4	1,94Na_2_O:Al_2_O_3_:0,84SiO_2_:194H_2_O:1TEA	100°C, 2 days	0.84*	4
LTA-9	1,94Na_2_O:Al_2_O_3_:0,84SiO_2_:194H_2_O:2TEA	100°C, 3 days	0.84*	9
LTA-22	1,94Na_2_O:Al_2_O_3_:0,84SiO_2_:194H_2_O:4TEA	100°C, 5 days	0.84*	22

^a^Si/Al ratios were measured from EDX; ^b^Particle sizes were measured from SEM images. ^*^Si/Al ratios in the gel.

The powdered sample of clinoptilolite with unit cell formula (Na_0*.*10_ K_0*.*57_)(Ca_0*.*47_ Mg_0*.*15_)(Al_1*.*97_Fe_0*.*12_)(Si_9*.*96_Ti_0*.*02_)O_24_ × 7H_2_O and the average particle size of 0.4 μm was obtained from Société Méditerranéenne des Zéolithes (Montpellier, France).

### Characterization of zeolite

The phase identification was accomplished using X-ray powder diffraction (XRD; Philips PW 1729 diffractometer, Ni-filtered Cu Kα radiation, Philips Electronics, Almelo, Netherlands). Crystal morphology/texture/surface features were examined by field emission scanning electron microscopy (FE-SEM; FEI 400 Quanta, FEI, Hillsboro, USA). Energy dispersive X-ray spectroscopy (EDX; Phoenix EDAX X-ray analyzer equipped with a Sapphire super ultra-thin window detector attached to a Hitachi S-4700 FE-SEM) was used to determine the crystal Si/Al ratios. Nitrogen adsorption-desorption isotherms were measured at the liquid nitrogen temperature using a Quantachrome Autosorb-6 analyzer (Quantachrome Instruments, Boynton Beach, USA). Prior to the measurements, samples were dried at 110°C for 12 h and outgassed at 300°C under high vacuum for 3 h. Multi-point Brunauer-Emmett-Teller (BET) specific surface areas (*S*_BET_) were calculated in the *P*/*P*_o_ range 0.05 to 0.30. The external specific surface areas (*S*_ext_) were estimated from the slope of the second linear segments on de Boer's adsorbate thickness *t*-plots. Saito-Foley (SF) method was applied for the pore volume and pore size analysis. The zeta potential of all samples was obtained by a zeta potential measurement system (Nano ZS90, Malvern Instruments, Worcestershire, UK) at 25°C. The particles were dispersed in deionized water (solid load is 1 wt%) and ultrasonicated for 1 h. After the ultrasonication, zeta potential was measured as a function of pH by titration with 0.1 M HCl or 0.1 M NaOH. The particle size distributions were measured on API Aerosizer LD equipped with API Aero-Disperser dry powder dispersion system (TSI, Inc., Shoreview, USA; Particle Instruments/Amherst, Amherst, USA). For these measurements, the density of zeolite Beta was assumed to be 1.61 g cm^−3^ [18]. Diffuse reflectance infrared Fourier transform (DRIFT) spectra were acquired on a Nicolet Magna-IR 560 spectrometer (Thermo Nicolet Corp., Madison, USA) supplied with a DTGS KBr detector (Thermo Nicolet Corp., Madison, USA) and a Spectra-Tech (Spectra-Tech, Inc., Oak Ridge, USA) diffuse reflectance high-temperature/vacuum chamber with KBr windows. The chamber loaded with crystals (0.02 g) was heated to 350°C for 2 h to dehydrate the samples. Subsequently, the spectra were collected 10 min after the samples were cooled to 100°C. The samples were under dry nitrogen (99.9% purity with <10 ppm moisture content, Med-Tech Gases, North Billerica, USA) flowing at 33 mL min^−1^ (STP) during the heat treatment in the chamber and during spectra acquisition. Before collection of the DRIFT spectrum, nitrogen was purged through the beam path at 14 L min^−1^ (NTP). The spectra were collected with resolution of 2 cm^−1^ using 128 scans. Potassium bromide (99 + % KBr, infrared grade, Acros Organics, Morris Plains, USA) was used as the background, and the samples were analyzed neat.

**Figure 1:**
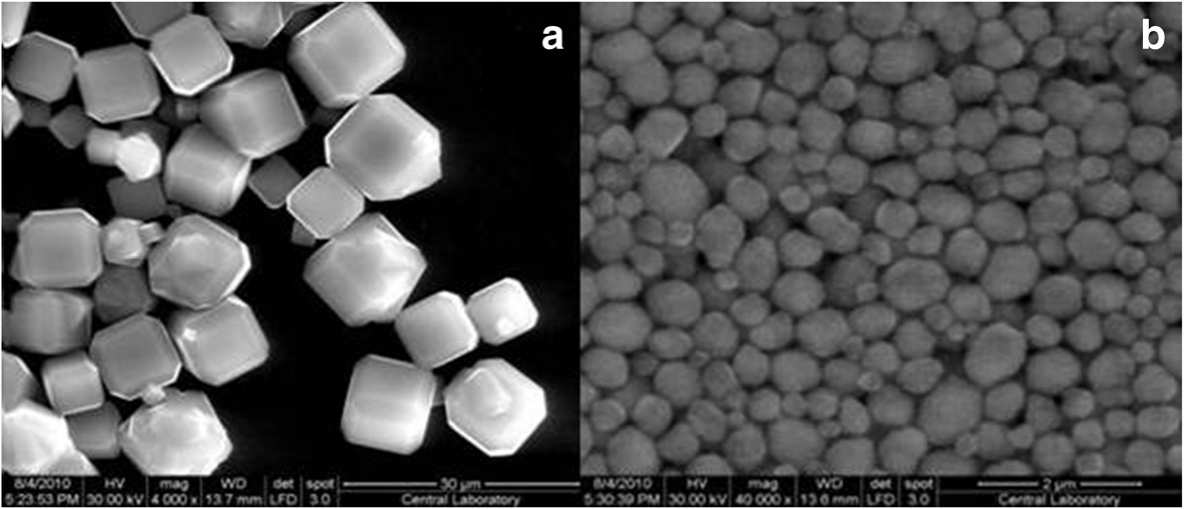
**Scanning electron microscopy images of synthesized LTA-9 (a) and BEA-40 (b) zeolites.**

The results of scanning electron microscopy images of synthesized LTA-9 and BEA-40 zeolite are presented in Figure 1.

As shown in Table 2, the BET characterization of as-synthesized zeolite BEA (Na^+^-BEA 30) showed that the pore volume, pore size, and surface area were 0.84 cc/g, 0.54 nm, and 260 m^2^/g, respectively. The modification of Na^+^-BEA 30 to NH_4_^+^-BEA 30 did not result in any significant changes in the results obtained from BET analysis as expected. However, the surface area (*S*_BET_, i.e., the total specific surface area), pore size, and pore volume of H^+^-BEA 30 was larger than that determined for the as-synthesized material. This increase in the BET surface area is likely due to an increase in the pore volume of zeolite Beta samples owing to the removal of structure directing agent from the pores upon heat treatment. Furthermore, the surface Si/Al ratio of the as-synthesized zeolite BEA 30 was found to be 10 ± 1 as measured by EDX. Ion exchanged and calcined samples prepared from the parent material showed the same value (10 ± 1).

**Table 2:** **Properties of zeolite samples**

**Sample name**	**Si/Ala**	**Surface area (m** ^2^ **/g)b**	**Pore size (nm)с**	**Pore volume (cc/g)c**	**Particle size (μm)**
Na^*+*^-BEA30	10 ± 1.0	260	0.54	0.84	Approximately 0.4
NH_4_^*+*^-BEA30	10 ± 1.0	279	0.64	0.89	Approximately 0.4
H^*+*^-BEA30	10 ± 1.0	490	0.85	1.3	Approximately 0.4
Na^*+*^-BEA50	17.7 ± 1.8	462	0.18	0.25	Approximately 0.4
NH_4_^*+*^-BEA50	17.7 ± 1.8	494	0.23	0.31	Approximately 0.4
H^*+*^-BEA50	17.7 ± 1.8	666	0.26	0.54	Approximately 0.4
LTA-4	0.84^d^	557	0.31	0.67	4
LTA-9	0.84^d^	550	0.28	0.51	9
LTA-22	0.84^d^	512	0.18	0.21	22

^a^Si/Al ratio of zeolite crystals was measured by EDX; ^b^Surface area of samples was measured by BET; ^c^Pore volume and size of the samples were measured by DR method; ^d^Si/Al ratios in the gel.

Linde type A (LTA) samples had almost the same surface area (500 to 550 m^2^/g) but differed in pore size, pore volume, and particle size. Si/Al ratio of all the LTA zeolites during synthesis was 0.84.

### Heat treatment of zeolite

Zeolite Beta crystals in the NH_4_^+^ form were obtained by ion exchange of the as-synthesized crystals with 1 M aqueous ammonium nitrate solution at 80°C for 24 h. The ion exchange procedure can be found elsewhere [19]. The ion-exchanged crystals (typically 0.2-g samples) were placed in a crucible and heated in a programmable furnace under ambient air to convert the NH_4_^+^ form of material into the H^+^ form having Brønsted acidity [19]. Sample BEA-1 was prepared by heating from 25°C to 500°C at a rate of 1°C min^−1^, maintaining at 500°C for 6 h, and then cooling to 25°C. Samples BEA-2 and BEA-3 were also treated using heat-up, isothermal, and cool-down steps but with different rates and temperature set points (Table 3).

**Table 3:** **Heat treatment procedures used to prepare different zeolite Beta samples**

**Sample name**	**Final temperature (°C)**	**Duration at final temperature (h)**	**Heating rate (°C min** ^**−1**^ **)**
BEA-1	500	6	1
BEA-2	700	6	10
BEA-3	700	6	1

### Sensor design

ISFETs were fabricated at the Institute of Semiconductor Physics (Kiev, Ukraine). Potentiometric sensor chip contains two identical *p*-channel transistors on the same crystal of total area 8 × 8 mm^2^. The diffusion *р*^*+*^-buses with contacts to the drain and source of each transistor are coming out on the edge of the chip along with an outlet to the built-in reference microelectrode. To prevent the formation of a parasitic conductivity channel between *p*^*+*^-areas of two transistors, the chip has a 50 μm wide protecting division *n*^*+*^-line with a contact to the substrate. Zigzag geometry of the transistor gate area with the width-to-length ratio 100:1 ensures sufficient steep slope of the transfer characteristic. The window etched in an oxide layer for growing gate dielectric replicates the channel geometry with a 7 μm overlapping.

The pH-FETs responses were measured using the circuit maintaining constant value of the current in the transistor channel, enabling the output signal to follow automatically the changes in potential value near the transistor gate. The threshold voltage for all pH-FETs was about −2.5 V. The measurements were performed under the following conditions: channel current of approximately 200 μA, drain-source voltage close to 1 V, and the sensor bulk is connected to the circuit ground. Transconductance of the used ISFETs measured in phosphate buffer with Ag/AgCl reference electrode was in the range of 300 to 400 μA/V. Typical current vs. pH response measured with standard buffer solutions (pH 4.0, 7.0, and 9.0) was in the range of 15 to 20 μA/pH, which gives us primary pH sensitivity estimation of approximately 50 mV/pH.

### Preparation of bioselective membranes

To produce a working bioselective membrane based on urease and zeolites, two mixtures were prepared. The first one contained 10% enzyme, 10% BSА, 10% glycerol in 20 mM phosphate buffer, pH 7.2, and the second - zeolite of different weight concentrations in 20 mM phosphate buffer, pH 7.2, with 10% glycerol. Prior to deposition on the transducer surface, the two mixtures were mixed in equal volumes. The final mixture was immediately deposited on the transducer surfaces using the Eppendorf microsampler (with variable volume 0.1 to 2.5 μL) till complete coverage of the working surfaces. The volume of each membrane was about 0.05 μL. The reference membrane mixture contained 10% BSA and 10% glycerol in 20 mM phosphate buffer, pH 7.2. All the membrane mixtures contained the same total amount of protein. Then, the transducers with deposited membrane mixtures were placed into glutaraldehyde saturated vapor for 15 to 25 min; afterwards, they were dried in the air at room temperature for 15 min and washed in the buffer solution from excess unbound proteins, zeolites, and GA.

### Measurement procedure

Measurements were carried out at room temperature in a working cell filled with 5 mM phosphate buffer, pH 7.2. The substrate concentrations were varied by addition of different portions of 500 mM urea solution to the cell. Every experiment was repeated three to six times for statistics. Non-specific changes in the output signal associated with fluctuations of temperature, medium pH, and electrical noise were avoided due to the usage of a differential measurement mode.

## Results and discussion

### Effect of parameters of BEA and LTA zeolites (crystal size, Si/Al ratio, and surface functional groups) on biosensor operation

**Figure 2:**
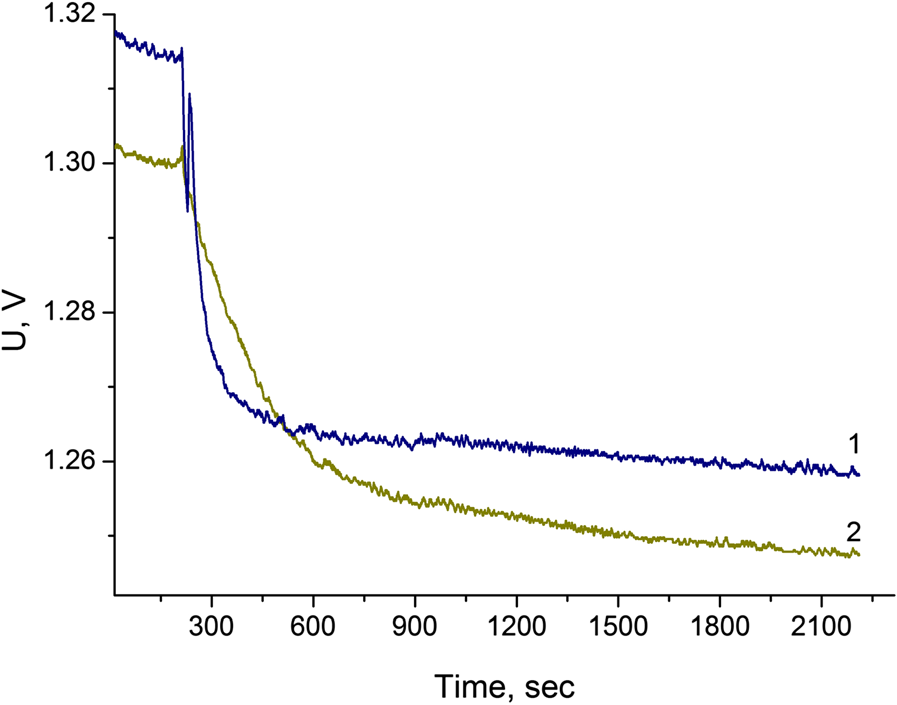
**Typical responses of biosensors based on urease (1) and urease with zeolite ES1 (2).** Measurements were conducted in 2.5 mM phosphate buffer, pH 6.4. Urea concentration - 3.5 mM.

The effect of addition of different types of zeolites with diverse particle sizes and Si/Al ratios on the analytical characteristics of urea biosensors based on ion-selective field-effect transistors was investigated. Zeolite BEA (BEA-30, 40, and 50) and zeolite A (LTA-4, 9, and 22) were used. The typical experimental responses to urea of urease-based biosensors and of biosensors based on urease with BEA-30 are presented in Figure 2. As seen, the response and the reaction rate of the biosensor without zeolite are faster as compared to the biosensor with zeolite BEA. This can be due to the fact that the zeolite particles could slow down the access of substrate to the enzyme within the biomembrane, and thus, the diffusion limitations become significant. However, the observed phenomena might lead to an increase in the linear range of urea measurement, which is another important parameter of biosensors. Thus, the extension of the linear range of urea determination with urease-based biosensors was considered as an item of further investigation.

**Figure 3:**
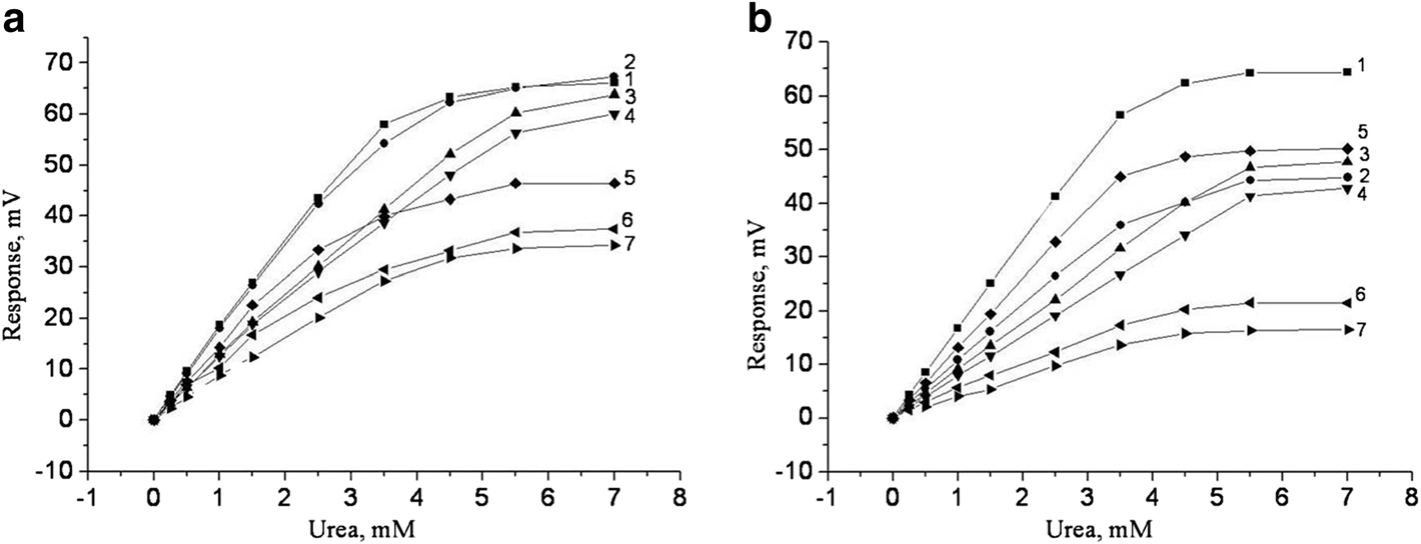
**Calibration curves of biosensors.** They were based on immobilized urease without zeolite (1) and urease with zeolite: BEA-30 (2), BEA-40 (3), BEA-50 (4), LTA-4 (5), LTA-9 (6), and LTA-22 (7). Zeolite concentrations are 1.5% **(a)** and 8.2% **(b)**. Measurements were conducted in 2.5 mM phosphate buffer, pH 6.4.

First of all, the characteristics of urease-based biosensors for direct urea determination were investigated as a function of zeolite weight percentage in the final membrane for different types of zeolites. Since sensitivity and linear range of measurement are two of the most important working characteristics of any biosensor, the effect of zeolite amount on these parameters was investigated. For this purpose, an array of urease-based biosensors was taken and different zeolites of various concentrations were added to each bioselective membrane. The calibration curves for each biosensor at each concentration (0.015, 0.15, 1.5, and 8.2) were plotted. It was seen that at low concentrations of zeolites (i.e., 0.015 and 0.15%), an increase in the zeolite concentration did not lead to a significant change in the responses for both zeolites LTA and BEA. At higher zeolite concentrations (i.e., 1.5 and 8.2%), a decrease in the sensitivity to urea was observed upon addition of zeolites, being more significant for LTA samples. At the same time, the linear range increased considerably, especially for BEA-40 and BEA-50 samples: 0 to approximately 3.5 mM and 0 to approximately 5 mM, respectively (Figure 3).

The observed more significant decrease in sensitivity to urea upon using LTA samples with respect to BEA samples can be due to a more hydrophilic nature of LTA samples (SiO_2_/Al_2_O_3_ ratio is approximately 1 for LTA and approximately 45 for BEA) and/or to noticeably bigger particle sizes (4 to 22 μm for LTA and 0.4 μm for BEA). The bigger particles have smaller surface area as in the case of LTA samples. Also, bigger zeolite particles could more efficiently prevent the substrate to reach the enzymatic sites. It seems that Si/Al ratio does not have a considerable effect on the biosensors in the current methodology of the biomembrane preparation. Possibly, the enzymes are not in close contact with the zeolites, and the zeolite particles act only like a diffusion barrier. Thus, the particle sizes might be more important for the biosensor sensitivity.

**Figure 4:**
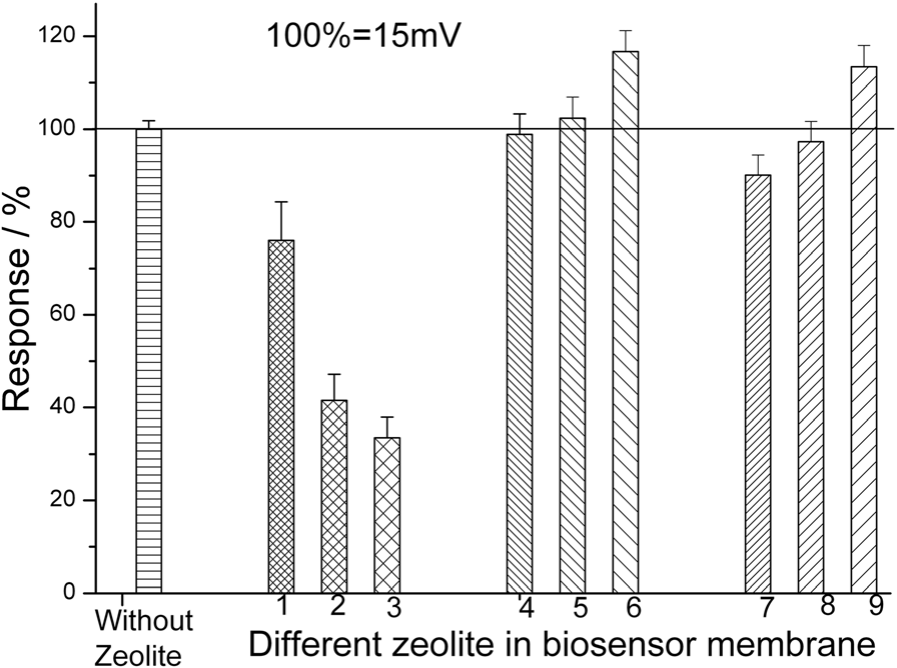
**Steady-state responses of urease biosensors without and with different zeolites in bioselective membranes.** (LTA-4 (1), LTA-9 (2), LTA-22 (3), BEA-30 (4), NH4-BEA-30 (5), H^+^-BEA-30 (6), BEA-50 (7), NH4-BEA-50 (8), and H^+^-BEA-50 (9)). Concentration of urea - 1 mM. Zeolite concentration - 5%. Measurements were conducted in 5 mM phosphate buffer, pH 7.2.

At the next stage, we investigated an application of different modifications of these zeolites for improving working characteristics of the urea-specific biosensors. For a start, we used zeolites LTA-4, LTA-9, LTA-22, BEA-30, NH_4_-BEA-30, H^+^-BEA-30, BEA-50, NH_4_-BEA-50, and H^+^-BEA-50. The linear ranges of measurement for biosensors based on the same zeolite with different surface modifications were similar, whereas their sensitivity differed. As shown in Figure 4, the biosensors with zeolites of LTA type (columns 1, 2, and 3) have the lowest sensitivity. It was revealed that the larger the size of LTA zeolite particle, the lower the biosensor sensitivity. The biosensors with BEA and NH_4_-BEA zeolites (columns 4, 5, 7, and 8) are characterized by approximately the same sensitivity as those based on urease only, except for the biosensors based on H^+^-BEA 30 and H^+^-BEA 50 (columns 6 and 9), which show higher sensitivity. It seems that an increase in hydrophilic properties of zeolites increases the biosensor sensitivity.

**Figure 5:**
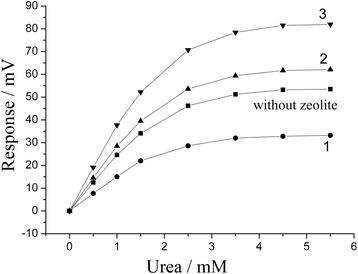
**Calibration curves of different urea biosensors.** They were based on: first (1), second (2), and third (3) variant of coimmobilization of urease with zeolite H^+^-BEA-30. Measurements were conducted in 5 mM phosphate buffer, pH 7.2.

An effect of slight increase in the biosensors sensitivity (about 20%) in the presence of zeolite H^+^-BEA-30 in the enzyme membrane was obtained at simultaneous immobilization of the enzyme together with zeolite in a mixture (one-layer immobilization). To enhance the positive effect, we attempted to perform two-layer immobilization. Three variants of the bioselective element construction were tested. The first one consisted of deposition of the enzyme membrane on the transducer surface (first layer) and the zeolite membrane as the second layer. In the second variant, the layers were placed *vice versa*. The third variant: first - zeolite layer and next layer - enzyme-zeolite mixture. A number of biosensors obtained by two-layer methods (all three variants) were examined; their calibration curves are shown in Figure 5. As seen, the biosensor with H^+^-BEA-30 zeolite immobilized according to the third variant had 50% higher activity than that of the biosensors with urease only.

### Effect of multilayer immobilization on function of clinoptilolite-based biosensors

At this stage, the main objective was to study the changes in characteristics of the biosensors upon addition of clinoptilolite to their bioselective elements.

**Figure 6:**
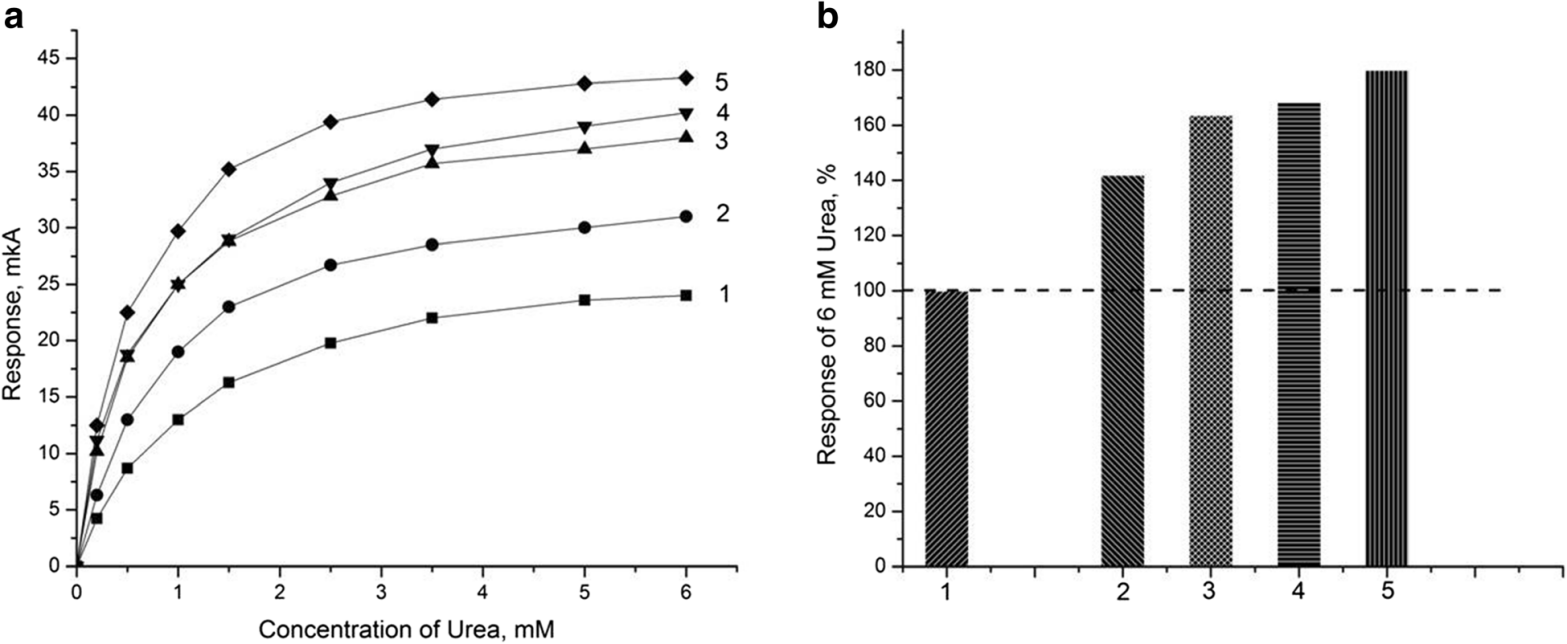
**Calibration curves (a) and sensor response to 6 mM urea (b).** Results were obtained in the absence of clinoptilolite (1). With addition of clinoptilolite of different concentrations to biomembranes: 1% (2); 5% (3); 10% (4); and 15% (5). Measurements were conducted in 5 mM phosphate buffer, pH 7.2.

The dependence of sensor response on the clinoptilolite concentration in the biological membrane was investigated. The calibration curves were plotted for the biosensors with different concentrations of zeolite in the membrane (Figure 6a). As seen, its increase results in a higher response (Figure 6b). At the zeolite concentration of 15% to 20%, a deterioration of the membrane adhesion to the transducer surface was observed as well as the membrane delamination and difficulties in the biomembrane drop-coating with a micropipette. It has been shown that there is no reason to add to the membrane more than 10% of zeolite.

In these experiments, an increase in the biosensor sensitivity was obtained using immobilization of the enzyme together with zeolite in a mixture. Further, we proposed two-layer immobilization using different combinations and ratios of zeolite and enzyme in the biomembrane. In the first case, 5% urease was deposited on the enzyme membrane and 10% BSA - on the reference membrane, and afterwards, immobilization was performed, and the sensor was washed to remove the GA excess; the second layers deposited on the same sensor were: 10% zeolite - on the enzyme membrane and 5% BSA - on the reference membrane.

**Figure 7:**
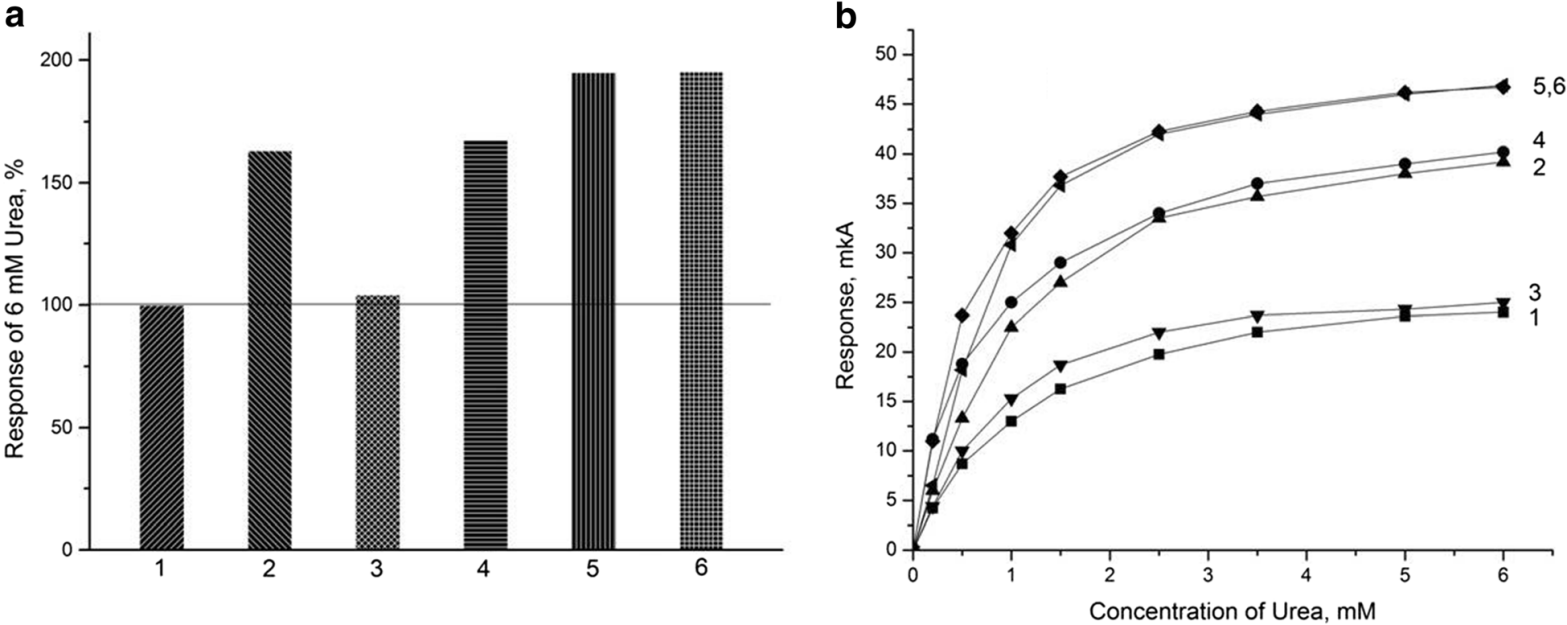
**Responses of urease biosensor to 6 mM urea (a) and calibration curves (b) for single- and two-layer membranes.** Single-layer urease membranes: 1 - with no zeolite; 2 - with a mixture of 5% zeolite and 5% urease. Two-layer membranes, which included: 3 - 5% urease in the first layer and 5% zeolite in the second layer; 4 - 5% zeolite in the first layer and 5% urease in the second layer; 5 - 5% zeolite in the first layer, a mixture of 5% zeolite and 5% urease in the second layer; 6 - 10% zeolite in the first layer, a mixture of 10% zeolite and 5% urease in the second layer; and 7 - a mixture of 5% urease and 5% zeolite in the first layer, a mixture of 5% urease and 5% zeolite in the second layer. Measurements were conducted in 5 mM phosphate buffer, pH 7.2.

In other case, the first layer as an enzyme membrane was zeolite, and the second layer - urease. The concentrations of all components remained the same as in the previous experiment. Next, bioselective element was prepared as follows. The first layer as an enzyme membrane was 5% zeolite, and the second layer - a mixture of 5% urease and 5% zeolite. As a reference membrane, the first layer was 5% BSA, and the second - 10% BSA. In the further experiments, the concentration of zeolite in the membrane was changed. In the first layer of the enzyme membrane, 10% zeolite was used, and in the second layer - a mixture of 10% zeolite and 5% urease, i.e., the enzyme membrane contained 20% zeolite. The BSA concentration in the reference membrane was not changed. Finally, both layers of the enzyme membrane contained a mixture of 5% zeolite and 5% urease. According to these schemes, a number of biosensors have been prepared and tested. Their calibration curves are presented in Figure 7.

As seen, when the enzyme is deposited on the sensor surface as the first layer and zeolite - as the second, the sensor sensitivity (curve 3) almost does not change compared to the urea sensor without zeolite (curve 1). Deposition of 5% zeolite as the first layer and 5% urease as the second (curve 4) increases the sensor responses by more than 1.5 times, and they are similar to the responses of the biosensors with single-layer membranes with a mixture of the enzyme and zeolite (curve 2). Deposition of 5% zeolite as the first layer and a mixture of 5% zeolite and 5% urease - as the second, results in even more significant increase in the biosensor responses (curve 5). However, a further increase of zeolite content in the membrane did not cause noticeable changes in the biosensor sensitivity (curve 6).

The results obtained can be explained by the fact that the presence of zeolite in the first layer improved diffusion properties of the membrane, which was a cause of higher sensitivity. Addition of zeolite in a larger concentration did not improve the sensor characteristics.

### Effect of BEA thermal modification on the functioning of biosensors in direct determination of substrates and inhibitory analysis of toxic substances

**Figure 8:**
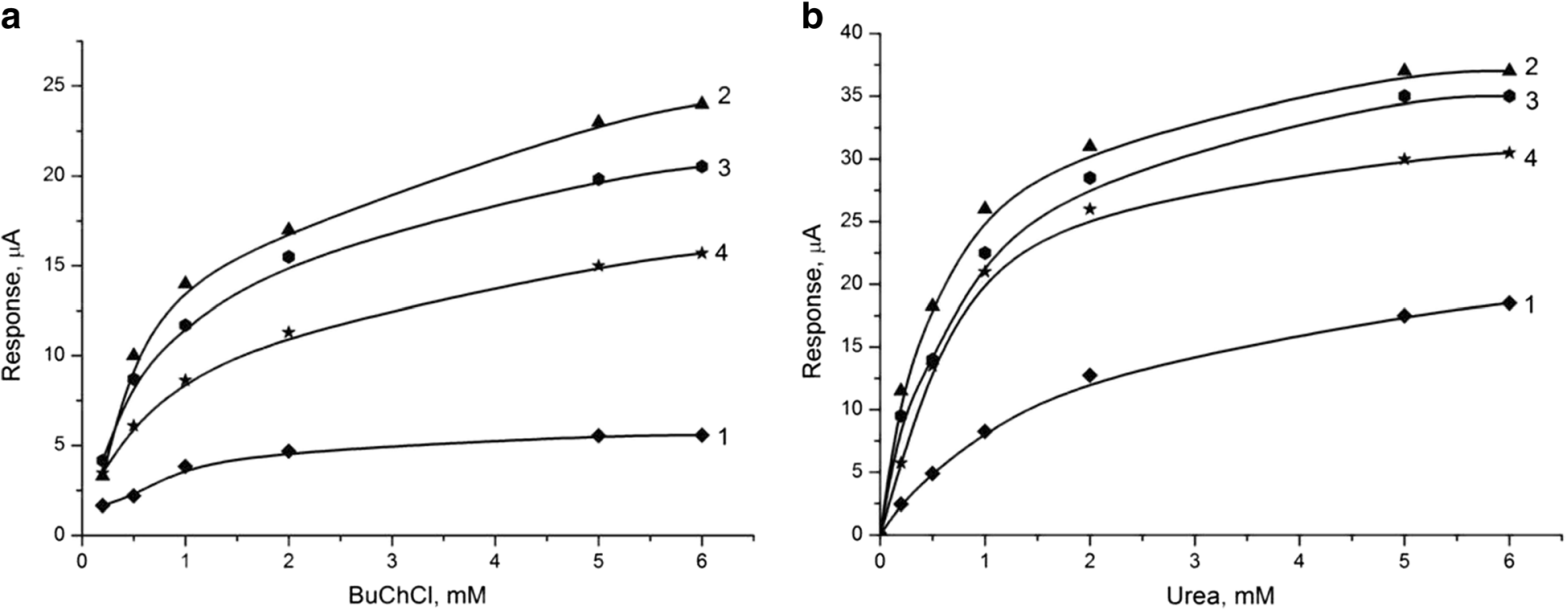
**Calibration curves of biosensors.** They were based on immobilized BuChE **(a)** and urease **(b)**, without zeolite (1) and with different heat-treated zeolite Beta: BEA-1 (1), BEA-2 (2), and BEA-3 (3). Measurements were conducted in 5 mM phosphate buffer, pH 7.4. The data shown represent the averaged response of five to seven different sensors.

At this stage of work, the characteristics of the zeolite-based biosensors were determined and compared depending on the type of heat treatment of zeolite (see section “Heat treatment of zeolite”). The calibration curves were plotted for the BuChE and urease biosensors modified by the heat-treated zeolite Beta particles. In general, zeolite addition to the enzyme membranes increased the responses for both types of biosensors. For both enzymes alike, the responses of biosensors modified with samples BEA-1 were the highest. Compared to the unmodified biosensors, these responses increased by up to approximately 5 times for BuChE and approximately 2 times for urease, as shown in Figure 8a,b, respectively. The biosensor responses obtained using samples BEA-2 and BEA-3 were lower, and the responses measured for all three zeolite Beta samples decreased in the order of BEA-1 > BEA-2 > BEA-3 for both biosensors investigated.

Before performing the experiments on application of the developed biosensors for inhibitory determination of toxic substances, it was necessary to confirm that a decrease in the biosensor response to the substrate observed after inhibition in the tested solution was due to the inhibition of the bioselective element and not a result of the error of measurement. For this purpose, operational stability, an important working characteristic of biosensors, was investigated. The biosensor responses to 5 mM BuChCl and urea were measured within one working day with 30-min intervals, during which ISFETs with immobilized biomembranes were kept in the working buffer solution. All biosensors based on zeolite/enzyme demonstrated high signal reproducibility. Additionally, the experiments on storage stability were performed. The biosensors were stored in the buffer solution at room temperature, and all samples containing zeolite Beta were stable for more than 5 days.

**Figure 9:**
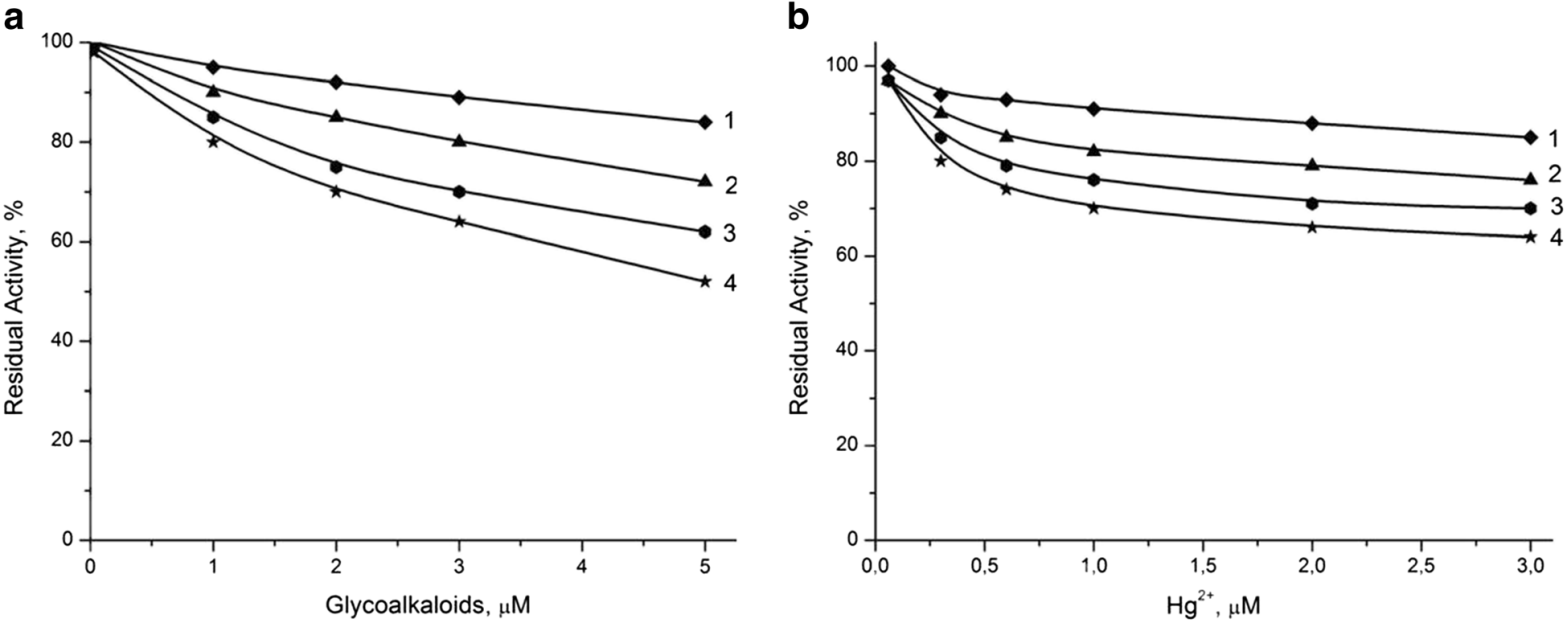
**Dependence of residual activity of bio-membranes.** They were based on BuChE **(a)** and urease **(b)** on concentration of glycoalkaloids and mercury ions (Hg + 2). Measurements were conducted in 5 mM phosphate buffer, pH 7.4. Figure legend: biosensor without zeolite (1) and with different heat-treated zeolite Beta: BEA-1 (1), BEA-2 (2), and BEA-3 (3).

The effect of different heat-treated zeolite samples incorporated in bioselective membranes on the biosensor sensitivity to glycoalkaloids and heavy metal ions was investigated using BuChCl and urea biosensors, respectively. The calibration curves of residual activity of biomembranes based on BuChE and urease as a function of the concentration of glycoalkaloids and mercury ions (Hg^2+^) are presented in Figure 9a,b, respectively. As shown in Figure 9a, all BuChCl biosensors based on zeolite Beta showed higher sensitivity to glycoalkaloids than the zeolite-free biosensor and these sensitivities decreased in the order of BEA-3 > BEA-2 > BEA-1. Similar trends were observed for the mercury ions in urea biosensors (Figure 9b).

## Conclusions

In the work presented, a number of potentiometric biosensors based on pH-ISFET and different types of zeolite have been developed. Their working characteristics have been determined and compared.

The biosensors based on BEA showed higher activity and better sensitivity to substrate compared with the biosensors based on LTA. The best results were obtained for BEA-40 and BEA-50, which are more hydrophobic than BEA-30. The biosensors based on zeolite BEA-40 and BEA-50 demonstrated also a certain increase in the dynamic range of urea determination. The obtained results indicate that Si/Al ratio and particle diameter influence the biosensor characteristics. As the Si/Al ratio increases, hydrophobicity increases too, and the dynamic range of the determination becomes wider. The possible explanation of this phenomenon is the formation of hydrophobic interaction between the enzyme and BEA samples that have higher Si/Al ratio. The particle diameter has a slight influence on the biosensor parameters.

LTA has relatively small pore sizes; thus, the enzyme molecules can interact only with an external surface of the zeolite particles. Since the LTA-4 and LTA-9 particles were bigger compared to LTA-22, the latter had lesser amount of domains to interact with the enzyme.

The working characteristics of potentiometric biosensors with clinoptilolite-containing membranes were also investigated. The optimum concentration of zeolites in bioselective elements, at which the highest biosensor sensitivity was observed, was found to be 10%. A variety of designs of bioselective elements with clinoptilolite were examined; the two-layer biomembrane (the first layer - 5% zeolite, the second - a mixture of 5% zeolite and 5% enzyme) was stated to be the most promising.

The surfaces of ISFETs were modified by incorporation of the heat-treated zeolite Beta crystals. The employed heat treatment procedures affected the amount of Brønsted acid sites, whereas the amount of terminal silanols in zeolite Beta did not change. Additionally, these procedures did not impact on the size, morphology, Si/Al ratio, and external specific surface area of zeolite Beta particles. The zeolite addition improved the biosensor sensitivity, and the responses increased with an increase of the zeolite Brønsted acidity. The inhibition characteristics obtained for the zeolite-modified biosensors also correlated with the amount of Brønsted acid sites created in the samples upon heat treatment. Thus, the results presented here show that the interactions between the enzymes and the Brønsted acid sites of a zeolite support can affect the actual biosensor performances.

These results show that it is possible to regulate the ISFET characteristics for different enzyme-based biosensors by tailoring the electrode surfaces via various zeolites. This makes zeolites strong candidates for integration into biosensors as ISFET modifiers.
